# Phosphocode-dependent glutamyl-prolyl-tRNA synthetase 1 signaling in immunity, metabolism, and disease

**DOI:** 10.1038/s12276-023-01094-x

**Published:** 2023-10-02

**Authors:** Eun-Young Lee, Jungwon Hwang, Myung Hee Kim

**Affiliations:** Microbiome Convergence Research Center, Korea Research Institute of Bioscience and Biotechnology (KRIBB), Daejeon, 34141 Korea

**Keywords:** Molecular biology, Biochemistry

## Abstract

Ubiquitously expressed aminoacyl-tRNA synthetases play essential roles in decoding genetic information required for protein synthesis in every living species. Growing evidence suggests that they also function as crossover mediators of multiple biological processes required for homeostasis. In humans, eight cytoplasmic tRNA synthetases form a central machinery called the multi-tRNA synthetase complex (MSC). The formation of MSCs appears to be essential for life, although the role of MSCs remains unclear. Glutamyl-prolyl-tRNA synthetase 1 (EPRS1) is the most evolutionarily derived component within the MSC that plays a critical role in immunity and metabolism (beyond its catalytic role in translation) via stimulus-dependent phosphorylation events. This review focuses on the role of EPRS1 signaling in inflammation resolution and metabolic modulation. The involvement of EPRS1 in diseases such as cancer is also discussed.

## EPRS1 signaling: a key player in immunity and metabolism

EPRS1, a vital enzyme for protein synthesis, has been found to play a significant role in various biological processes beyond translation. Researchers have discovered that EPRS1 can act as a molecular switch, regulating inflammation, metabolism, and disease. The enzyme’s phosphorylation at specific sites allows it to dissociate from the multi-tRNA synthetase complex (MSC) and interact with different proteins to perform various functions. For example, EPRS1 regulates inflammation resolution in response to LPS and bacteria, adipocyte lipid metabolism in response to insulin, and antiviral immune function in response to RNA viruses. These findings highlight the potential of EPRS1 as a therapeutic target for treating inflammatory diseases, obesity, aging, and viral infections. Further research is needed to better understand the mechanisms underlying EPRS1’s diverse functions and develop targeted therapies.

## Introduction

A healthy human body requires the maintenance of a finely tuned homeostatic balance via systemically controlled biological processes. Aminoacyl-tRNA synthetases (ARSs) are essential for protein synthesis because they charge specific transfer ribonucleic acids (tRNAs) with their cognate amino acids (i.e., tRNA aminoacylation) at the preribosomal stage of translation^1,2^. Compared with their prokaryotic equivalents, eukaryotic ARSs have acquired additional motifs and domains through evolution that enable their eukaryote-specific functions in addition to translation^3–5^. Research over many decades has assumed that abundant and ubiquitously expressed ARSs are intrinsic crossover mediators of biological processes that sustain homeostasis, mainly through their additional evolutionarily acquired motifs and domains^5–7^.

The nomenclature of eukaryotic ARSs is based on single-letter abbreviations corresponding to their substrate amino acids, followed by “ARS1” for cytoplasmic ARSs and by “ARS2” for their mitochondrial counterparts. The human genome encodes eight cytoplasmic ARSs: DARS1, EPRS1 (the only bifunctional tRNA synthetase), IARS1, KARS1, LARS1, MARS1, QARS1, and RARS1, together with three scaffold proteins called aminoacyl-tRNA synthetase-interacting multifunctional protein 1 (AIMP1), AIMP2, and AIMP3. These proteins form a complex known as the multi-tRNA synthetase complex (MSC) via their appended motifs and domains^6,7^. Genetic depletion of the core scaffold protein AIMP2 from the MSC in mice resulted in lethality but did not change the global protein synthesis rate or cell viability^8^, suggesting that the structural integrity of the MSC is essential for life (at the systemic level) but may not be necessary at the cellular level. Although the mechanism by which this complex is assembled and maintained or how its functions are coordinated with other biological processes remain unclear, it clearly functions as a molecular hub for ARS-coordinated protein synthesis and their noncanonical functions in a stimulus- or context-dependent manner^3,6,7,9^. Under normal conditions, an ARS is almost exclusively part of an MSC but not an individual ARS^10,11^. One study that compared the signal generated on a blot with a standard curve reported that the average copy number of KARS1 was 10^7^ per cell, which is equivalent to 9 × 10^6^ assembled MSCs per cell^11^. ARSs are closely associated with the MSC to preserve a stable architecture^10^. However, changes in cell state or context or homeostasis disruption lead to a switch in ARS function from translation to noncanonical signaling via posttranslational modification (most often phosphorylation)-mediated dissociation from the MSC^12^.

Among MSC components, EPRS1 is the most profound, representative player that plays multiple roles in maintaining homeostasis. It comprises EARS1 and PARS1, which are coupled via a linker consisting of three WHEP domains named after a subset of ARSs (WARS1 (W), HARS1 (H), and EPRS1 (EP))^13,14^. The advantage of the specific selection of two ARSs for fusion is not known. One study suggested the possibility that the unique metabolic relationship between glutamic acid and proline may be the underlying factor that drives the fusion of two cognate ARSs^15^. Studies from the past two decades collectively suggest that EPRS1 functions as a molecular switch that triggers multicellular functions, including immune and metabolism activities, in a stimuli-dependent manner^16–20^. In this review, we discuss the context-specific role of EPRS1 signaling in immunity, metabolism, and disease, along with its role in particular cell types, physiological states, and environments.

## Distinctiveness of the Eprs1 structure

**Fig. 1:**
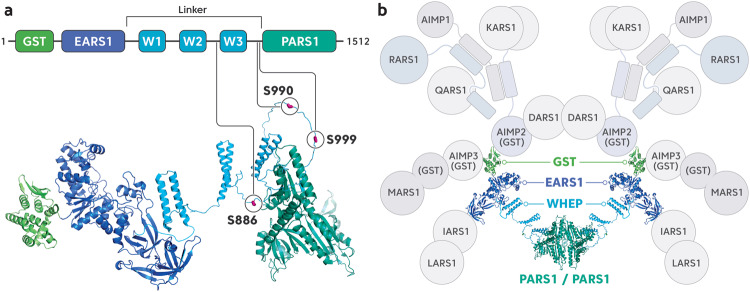
The domain structures of EPRS1 and its involvement in the MSC. **a** Schematic diagram (upper panel) and linearly displayed structures of the domains within EPRS1 (lower panel). GST, glutathione-S-transferase-like domain; EARS1, glutamyl-tRNA synthetase 1; Linker, the region comprising three WHEP domains; W, WHEP domain; PARS1, prolyl-tRNA synthetase 1. Phosphorylation sites (Ser886, Ser990, and Ser999) within the Linker are indicated. The structures of each domain are displayed using the crystal structures of GST (green, PDB ID 5A57) and PARS1 (emerald green, PDB ID 4K86), as well as the NMR-based structure of W1 (sky blue, PDB ID 1FYJ). EARS1 (navy blue), W2 (sky blue), and W3 (sky blue), which were modeled using AlphaFold2^103^ combined with experimentally determined structures. The EARS1 structure was predicted with high confidence according to the estimated per residue confidence score (pLDDT > 80). The helix-turn-helix W2 and W3 domains were modeled with high confidence (pLDDT > 80), while the disordered regions between W1 and PARS1 were modeled with low confidence (pLDDT < 50). S886, S990, and S999 are indicated. **b** Cartoon representation of a human MSC based on published data^6^ and structural information^21,104–106^. An MSC comprises EPRS1 (a dimer), DARS1 (a dimer), KARS1 (a dimer), RARS1, QARS1, MARS1, IARS1, LARS1, AIMP1, AIMP2, and AIMP3. Dimeric EPRS1 within the MSC is shown in a ribbon diagram. Leucine zippers are represented by cylinders.

Human EPRS1 is a 1512 amino acid-long bifunctional tRNA synthetase (Fig. 1a) that resides exclusively in the MSC (Fig. 1b). Due to its physical flexibility, which correlates with its multicellular functions, the overall structure of EPRS1 has not yet been determined. However, the structures of the glutathione-S-transferase-like (GST) domain^21^, the first WHEP domain (W1)^22^, and PARS1^23,24^ have been solved via X-ray crystallography and nuclear magnetic resonance (NMR) spectroscopy. In EPRS1, EARS1 and PARS1 are connected by a long linker region (Linker) of approximately 300 amino acids, which comprises triple repeats of the 50-amino-acid WHEP domain, which adopts a helix-turn-helix structure. The Linker functions as a noncanonical functional constituent of EPRS1. The N-terminal GST domain of EPRS1 is associated with other GST domains within the MSC (Fig. 1b). Indeed, it interacts directly with the GST domains of the auxiliary proteins AIMP2 and AIMP3; the AIMP3 GST domain binds to the MARS1 GST domain to form a tetrameric GST complex that is generated in the sequential order MARS1–AIMP3–EPRS1–AIMP2 in an MSC^4^. These GST domains are critical for maintaining the structural integrity of the MSC.

ARSs are classified into two groups (Class I and Class II ARSs) based on their structural features. The class to which an ARS belongs depends on its catalytic core structure, which is the site of ligation reactions. The active sites of Class I ARSs are formed by a Rossmann nucleotide-binding fold carrying a parallel β-sheet harboring two highly conserved HIGH and KMSKS motifs that bind to a tRNA acceptor stem from the minor groove side^25–29^. In contrast, the active site of a Class II ARS is formed at the core of the antiparallel β-strands that bind to the major groove side of the acceptor stem via three conserved sequence motifs^30,31^. Consistent with these different approaches to the tRNA acceptor stem, Class I and Class II ARSs catalyze the aminoacylation of different hydroxyl groups on the adenosine of a terminal tRNA; Class I ARSs attach an amino acid to the 2’-OH of a tRNA terminal nucleotide, whereas Class II ARSs attach an amino acid to the 3’-OH^32,33^. Most Class I ARSs are monomeric, whereas most Class II ARSs are dimeric^30,34,35^. Since the PARS1 domain within EPRS1 is a Class II enzyme, EPRS1 forms a dimeric structure. This dimeric conformation is critical for EPRS1 involvement in maintaining the twofold symmetry of an MSC, together with dimeric DARS1 (Fig. 1b)^6,7^. PARS1 does not appear to contact any other MSC components, whereas EARS1 interacts directly with IARS1^36^.

**Fig. 2:**
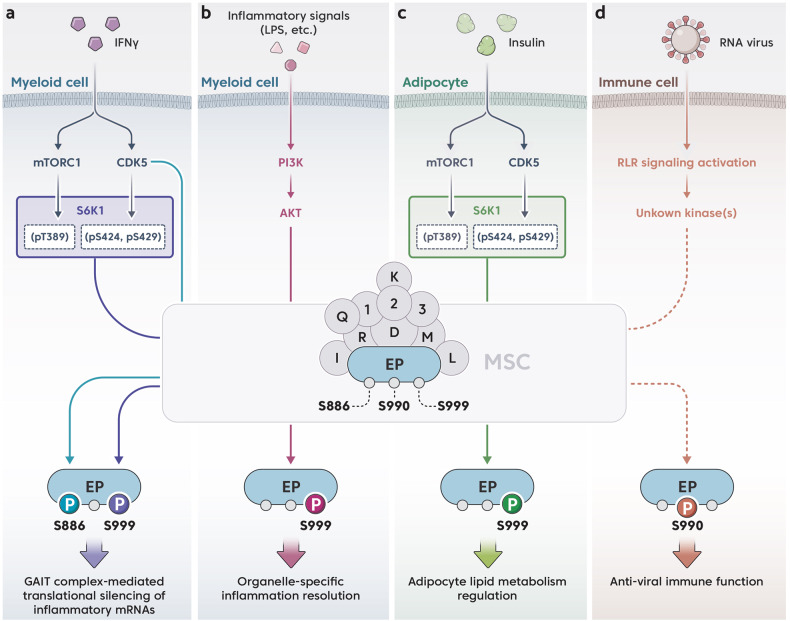
Phosphocode-dependent EPRS1 signaling pathways are activated by different stimuli. **a** IFN-γ treatment activates the CDK5 and mTOR/S6K1 pathways, which sequentially phosphorylate EPRS1 at Ser886 and Ser999 in myeloid cells. Modified EPRS1 is released from an MSC and forms a GAIT complex to silence the expression of inflammatory genes. **b** Inflammatory TLR ligands activate the PI3K/AKT pathway in myeloid cells to trigger EPRS1 phosphorylation at Ser999, resulting in the release of EPRS1 from MSCs. Released EPRS1 coordinates with an organelle-specific AKT signaling complex to resolve inflammation. **c** Insulin-mediated noncanonical multisite phosphorylation of S6K1 in adipocytes induces EPRS1 phosphorylation at Ser999. Modified EPRS1 is released from an MSC to regulate lipid metabolism. **d** Upon viral entry into cells, RIG-I is activated and initiates an antiviral signaling cascade that induces the phosphorylation of EPRS1 at Ser990 by an unidentified kinase(s). Modified EPRS1 is released from an MSC to positively regulate antiviral immune responses. The MSC is composed of the following components: EP, EPRS1; D, DARS1; K, KARS1; R, RARS1; Q, QARS1; M, MARS1; I, IARS1; L, LARS1; 1, AIMP1; 2, AIMP2; and 3, AIMP3.

**Fig. 3:**
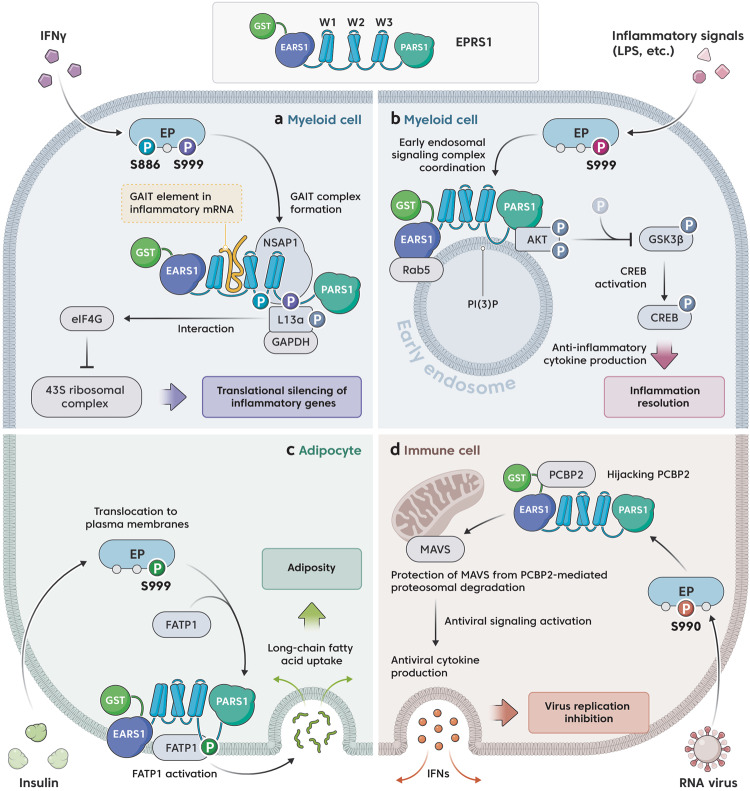
Mechanistic actions of EPRS1 released from MSCs. **a** In myeloid cells, EPRS1 modified at Ser886 and Ser999 forms a heterotetrameric GAIT complex with NSAP1, P-L13a, and GAPDH in response to IFN-γ stimulation. Within this complex, EPRS1 binds to GAIT-element-containing inflammatory mRNA through its W1 and two other domains in the Linker, while L13a interacts with eIF4G in the translation initiation complex, thereby blocking recruitment of the 43S ribosomal complex subunit and repressing translation. **b** TLR-mediated inflammatory signals in myeloid cells induce the association of EPRS1 (modified at Ser999) with the early endosomal membrane via a PI(3)P-binding motif in the Linker. The EPRS1-mediated trafficking of AKT to the early endosome results in the assembly of an endosome-specific AKT signaling complex containing Rab5. This complex promotes AKT-mediated GSK3β phosphorylation, which increases CREB activation and anti-inflammatory cytokine production to maintain physiological homeostasis during inflammation. **c** Insulin-induced modification of EPRS1 at Ser999 in adipocytes regulates lipid metabolism via the translocation of FATP1 to the plasma membrane, followed by its activation. This event stimulates the long-chain fatty acid uptake required for increased triglyceride synthesis, leading to adiposity. **d** After RNA virus infection, EPRS1 modified at Ser990 is released from the MSC and hijacks PCBP2 to stabilize MAVS, which functions as a central hub for antiviral signaling in immune cells. EPRS1 competes with PCBP2 for binding MAVS, thereby blocking the PCBP2-mediated ubiquitination and proteasomal degradation of MAVS. This event promotes the production of the antiviral cytokine type I IFN, thereby suppressing viral replication. Although EPRS1 forms a dimer via its PARS1 domain, it is illustrated as a monomer for clarity.

EPRS1 is phosphorylated at Ser886, Ser990, and Ser999 in the Linker (Fig. 1a). These modifications are prerequisites for EPRS1 dissociation from the MSC to perform multicellular functions in response to various stimuli (Figs. 2 and 3)^16–20^.

Based on information obtained from the PhosphoSitePlus database (http://www.phosphosite.org), it has been predicted that approximately 60 of the 1512 amino acids in EPRS1 can be phosphorylated^37^; however, only three phosphorylation sites (i.e., Ser886, Ser990, and Ser999) have been validated using specific phospho-antibodies and mass spectrometry analysis^16–20^. Recently, phosphomimetic Ser-to-Glu mutations in EPRS1 (S688E and S691E) revealed the dissociation of EPRS1 from IARS1 in the MSC complex in cells^36^. However, this study did not use specific antibodies to validate the phosphorylation status of these residues. Thus, in this review, we focus on the roles of the Ser886, Ser990, and Ser999 residues in multicellular functions.

## Resolution of inflammation

Inflammation is a biological defense mechanism against infection or sterile tissue damage. In response to inflammatory challenges, immune cells actively produce cytokines and chemokines to trigger systemic responses that ultimately restore homeostasis^38^. Uncontrolled or unresolved inflammation is a key driver in the progression of chronic diseases, resulting in detrimental disorders such as cancer^39–41^. To limit the undesirable consequences of excessive inflammatory responses, many mediators trigger signaling pathways that actively resolve inflammation^41–43^. Recent studies have revealed that the housekeeping enzyme EPRS1 functions as a mediator of inflammatory homeostasis via a posttranscriptional off switch^10,44^ or by coordinating organelle-specific anti-inflammatory signaling complexes^20^. Defects in the inflammation-resolving functions of EPRS1 accelerate the progression of the inflammatory diseases described in the following sections.

### GAIT complex-mediated silencing of inflammatory genes

The first noncanonical function of EPRS1 was discovered two decades ago in a study showing that EPRS1 executes translational silencing of specific genes^10^. After stimulation by IFN-γ, which is a key event in inflammation initiation^45^, EPRS1 dissociates from the MSC in a phosphorylation-dependent manner (Fig. 2a). The free EPRS1 interacts with NS1-associated protein 1 (NSAP1) to form a nonfunctional ‘pre-γ-interferon-activated inhibitor of translation (pre-GAIT) complex’. Then, the ribosomal protein L13a (phosphorylated at Ser77) and glyceraldehyde‐3‐phosphate dehydrogenase (GAPDH) are recruited to the pre-GAIT complex to complete the assembly of the functional heterotetrameric GAIT complex (Fig. 3a). The GAIT complex inhibits the translation of specific mRNAs that bear RNA hairpins, known as GAIT elements, in their 3’ untranslated regions (3’ UTRs)^10,46^. The GAIT complex silences the translation of ceruloplasmin (Cp), a protein linked to inflammatory responses^10^. In addition to Cp, other GAIT-element-containing genes have been identified, and several of these (including mRNAs encoding multiple chemokine ligands and receptors) are associated with genes encoding proteins involved in inflammation^47–49^. Translation silencing contributes to the inhibition of inflammatory responses by infiltrating or resident macrophages following cytokine activation. Through its W1 and 2 domains in the Linker, EPRS1 is the only protein within the heterotetrameric complex that binds directly to the GAIT RNA element. The upstream pair of WHEP domains facilitates high-affinity binding to GAIT-element-bearing mRNAs. The downstream WHEP pair (i.e., W2 and 3) is involved in EPRS1 interaction with NSAP1 in the pre-GAIT complex, which inhibits mRNA binding. The interaction between EPRS1 and L13a/GAPDH triggers conformational changes that allow W1 and 2 to bind to the GAIT element in target mRNAs to facilitate translational silencing (Fig. 3a)^13^.

Since the discovery of the EPRS1 mechanism of GAIT-element binding, the mechanism underlying GAIT action was precisely elucidated. A proteomic approach was used, and specific EPRS1 phosphorylation sites critical for GAIT complex formation were identified. After 1 and 2 h of IFN-γ stimulation, EPRS1 is sequentially phosphorylated at Ser886 and Ser999 in the Linker; the phosphorylation rate was highest within 4 h^16^. Phosphorylation of EPRS1 at Ser886 is required for EPRS1 interaction with NSAP1 and formation of the pre-GAIT complex, whereas phosphorylation of Ser999 directs the formation of the functional GAIT complex that binds to eukaryotic initiation factor 4G (eIF4G) to block ribosome recruitment, leading to repressed GAIT gene translation (Fig. 3a). Phosphorylated EPRS1 is not incorporated into an MSC, and Ser999 phosphorylation is essential for the stable dissociation of EPRS1 from the complex^16^. Notably, Ser886 is not conserved in mouse EPRS1 (the corresponding residue encodes asparagine not serine). Therefore, in contrast to its modification in humans, EPRS1 is phosphorylated only at Ser999 not at Ser886. As a result, mouse EPRS1 associates with the heterotrimeric GAIT complex along with L13a and GAPDH, although NSAP1 is not included in the complex^50^. These results suggest that Ser999 phosphorylation is the key event that confers structural and functional pliability to EPRS1 allowing it to governing of inflammatory gene silencing.

Distinct kinases are critical for EPRS1 phosphorylation (Fig. 2a)^17,19^. The first event leading to GAIT assembly is IFN-γ-mediated activation of cyclin-dependent kinase 5 (CDK5) in conjunction with its activator, p35; activated CDK5/p35 directly phosphorylate Ser886 in EPRS1^17^. The kinase axis of the mammalian target of rapamycin complex 1 (mTORC1) and p70 ribosomal protein S6 kinase 1 (S6K1) also contributes to Ser999 phosphorylation^19^. Ser999 phosphorylation requires CDK5-mediated phosphorylation of S6K1 (at Ser424 and Ser429)^51^. CDK5 inhibition blocks these phosphorylation events, which prevents EPRS1 release from an MSC and abrogates EPRS1 functions in the GAIT complex, leading to increased expression of inflammatory proteins^17^.

Diverse forms of EPRS1 have been identified in cells. One study revealed that a novel polyadenylation event, which introduces a new stop codon, generates an EPRS1 variant with a truncated C-terminus called EPRS1(N1)^52^. This truncated form of EPRS1 preserves a homeostatic basal level of GAIT gene expression in myeloid cells, thereby playing an important role in cellular homeostasis by inhibiting the binding of GAIT to target mRNAs. For example, although vascular endothelial growth factor A (VEGF-A) is an angiogenic factor that supports tumor growth, it is essential for vessel maintenance, indicating a requirement for its basal expression for homeostasis maintenance. A study showed that the GAIT complex repressed VEGF-A synthesis, which proceeded at a low constant rate independent of VEGF-A mRNA levels. The EPRS1(N1) truncation mutant shields GAIT-element-bearing transcripts (through high-affinity binding to GAIT target mRNAs) from the inhibitory GAIT complex, thereby maintaining low-level expression of GAIT target proteins (e.g., VEGF-A).

In addition, proteolytic cleavage of EPRS1 by caspases has been demonstrated^53^. Caspases are cysteine-dependent aspartic acid proteases that cleave proteins after aspartate residues^54^. EPRS1 is cleaved at the third WHEP domain, which carries a highly conserved cleavage site, ^926^DQVD^929^. Calcium-activated calpains also target EPRS1 at several undefined sites, including the WHEP domains, and proteolytically cleave EPRS1 into fragments^55^. It is unclear whether cleaved EPRS1 fragments lacking part of the targeted WHEP domain inhibit GAIT function. It is thought that several forms of EPRS1 are required for the inactivation of unknown noncanonical functions or for the generation of bioactive fragments with distinct noncanonical activities^53^.

In summary, the GAIT system potentially fine-tunes inflammatory gene expression in the presence of persistent inflammatory stimuli and facilitates inflammation resolution after the stimulus is removed^44^. The WHEP domain-containing Linker is harbored in most metazoan EPRS1 molecules, implying a substantial evolutionarily conserved benefit^13^. In higher vertebrates, the regulatory mechanism controlled by the GAIT complex seems to involve a machinery that prepares the organism to resolve exacerbated inflammation caused by infection, illness, or stress.

### Organelle-specific anti-inflammatory functions

Recently, a novel mechanism by which EPRS1 coordinates the anti-inflammatory signaling complex in subcellular organelles was identified^20^. The phosphoinositide 3-kinase (PI3K)/AKT pathway inhibits proinflammatory responses in lipopolysaccharide (LPS)-stimulated macrophages^56^. In particular, the AKT signaling pathway regulates Toll-like receptor (TLR) 4 hypersensitivity in the myeloid cell lineage by inhibiting the expression of inflammatory mediators^57,58^. During this process, AKT is activated via inflammatory stimuli and is translocated to the plasma membrane and intracellular membrane compartments^59^. The authors found that EPRS1 exhibited crucial anti-inflammatory roles by recruiting activated AKT to early endosomes to regulate the downstream target glycogen synthase kinase 3β (GSK3β)^20^. In early endosomes, AKT increased GSK3β phosphorylation at Ser9 and inhibited its activity, which then reduced inflammatory cytokine production by monocytes^60,61^.

Specifically, EPRS1 is phosphorylated at Ser999 after monocytes are stimulated via TLR2, TLR4, or TLR9 ligand binding to cognate receptors; phosphorylation of Ser99 drives EPRS1 dissociation from an MSC (Fig. 2b)^20^. The TLR3 ligand poly(I:C) does not affect Ser999 modification, suggesting that myeloid differentiation primary response 88 (MYD88) is required for EPRS1 function. Phosphorylation at Ser999 in monocyte/macrophage cells under inflammatory conditions has been observed as early as 15 min after stimulation, peaking at 60 min; these kinetics are consistent with the dissociation of EPRS1 from MSCs. Notably, TLR ligands did not trigger the production of IFN-γ in monocytes/macrophages, indicating that EPRS1 phosphorylation at Ser999 is independent of IFN-γ, which triggers GAIT complex formation. An interaction study revealed that EPRS1 interacted specifically with AKT (functioning as a proximal kinase) under inflammatory conditions. Kinase inhibitor assays confirmed that AKT was critical for the phosphorylation of EPRS1 at Ser999 (Fig. 2b).

After its release from MSCs, EPRS1 continues to interact with AKT and traffics AKT to early endosomes via a phosphatidylinositol-3-phosphate (PI(3)P)-binding motif (also found in the CBR3 loop of the noncatalytic C2 domain in phosphatase and tension homolog (PTEN)^62^) located between the third WHEP domain and PARS1 domain (Fig. 3b). Mutation of a consensus sequence in the (PI(3)P)-binding motif abolished the interaction between EPRS1 and PI(3)P. Further interaction analysis revealed that EPRS1 was associated with Rab5B and Rab5C in addition to AKT, implying early endosomal trafficking. Signaling by the GTP-bound activated form of Rab5/PI(3)P on early endosomal membranes recruits effector proteins^63–65^. The surface area of endomembranes is up to 200-fold that of the plasma membrane^66^. These endosomal membranes are physical platforms where specific signaling complexes are assembled^67,68^. As the PI3K/AKT pathway controls multiple downstream cellular processes, this axis must be intricately regulated to target specific functions^69,70^. Therefore, EPRS1-mediated intracellular partitioning of AKT to endosomal membrane compartments governs AKT substrate specificity, which also ensures that AKT activity level remains proportional to the level of sustainable inflammation-stimulating signals. This unique feature of EPRS1 might be critical for maintaining immunological homeostasis for extended periods and might provide clues regarding sustainable AKT activation in the cytosol.

Domain assignment for the EPRS1 interaction revealed that EARS1 participates in the interaction with Rab5, while PARS1 is critical for AKT binding (Fig. 3b). As the PARS1 domain is adjacent to Ser999 located in the Linker (only 24 amino acids separate them), PARS1 may harbor a docking site for AKT that positions the AKT kinase domain proximate to the EPRS1 Ser999 residue. Moreover, the N-terminal pleckstrin homology (PH) domain of AKT mediates autoinhibition by blocking the substrate-binding sites in the kinase domain, thereby promoting AKT dephosphorylation^71^. EPRS1 may maintain AKT in an active conformation by interacting with its kinase domain; alternatively, PH domain binding may interfere with AKT activation. In summary, inflammatory stimulation-dependent MSC-dissociated phospho-EPRS1 is translocated to early endosomes to promote the assembly of an endosome-specific anti-inflammatory AKT signaling complex: the PARS1 domain traffics AKT to early endosomes via the PI(3)P-binding motif in the Linker, and the EARS1 domain interacts with Rab5 (Fig. 3b).

AKT activation in EPRS1-deleted bone marrow-derived macrophages (BMDMs) decreased significantly following exposure to inflammatory stimuli (i.e., LPS), resulting in decreased AKT-mediated phosphorylation of GSK3β Ser9. Phosphorylation of GSK3β at Ser9 inhibited GSK3β activity, which in turn triggered anti-inflammatory cytokine production and negatively regulated NF-kB-mediated signaling via the activation of cAMP response element-binding protein (CREB)^72–75^. Thus, EPRS1 deficiency in BMDMs markedly reduced CREB activation while activating NF-kB, leading to increased levels of proinflammatory cytokines (TNF-α and IL-6) and attenuated anti-inflammatory cytokine (IL-10) production^20^.

Three representative mouse models have been established to validate the in vivo role of EPRS1 under inflammatory conditions: (i) mice with LPS-mediated endotoxic shock; (ii) mice infected with the pathogen *Salmonella Typhimurium*; and (iii) mice treated with dextran sulfate sodium (DSS) to induce colitis, which establishes these mice as models of intestinal inflammatory conditions^20^. EPRS1-deficient mice were found to be more susceptible to LPS-induced septic shock and *S. typhimurium* infection. The levels of anti-inflammatory (IL-10) cytokines were significantly lower in EPRS1-deficient mice than in wild-type mice, whereas the levels of proinflammatory cytokines were higher. Histological studies revealed that inflammatory cell infiltration of the lungs was more extensive in EPRS1-deficient mice. Moreover, in EPRS1-deficient colitis mice, body weight loss, disease activity scores, and damage scores were higher than they were in wild-type (WT) colitis model mice. Collectively, these results demonstrate that EPRS1 is a critical effector protein that resolves inflammation to preserve physiological homeostasis^20^.

Under inflammatory conditions, EPRS1 seems to play a compensatory role in resolving inflammation by coordinating the action of the anti-inflammatory AKT signaling complex^20^, as well as by assembling the GAIT complex^16^. However, in contrast to EPRS1/AKT-driven anti-inflammatory immune regulation, which is triggered within 1 h of stimulation, functional GAIT complexes are assembled approximately 24 h following IFN-γ stimulation. Although TLR activation in macrophages did not generate IFN-γ in vitro, both the GAIT complex and the EPRS1/AKT-mediated signaling complex contributed to the resolution of inflammation in vivo. Physiologically, the first line of anti-inflammatory immune defense is likely directed via the TLR/PI3K/AKT-mediated activation of EPRS1, whereas the GAIT system is more likely to resolve chronic and persistent inflammation.

## Modulation of metabolic functions

Because inflammation is a common denominator in age-associated pathologies such as metabolic syndromes and diabetes^76^, there is no doubt about the role of EPRS1-mediated functions in metabolism. The clues are derived from the role played by EPRS1 in adipocytes, in which it is phosphorylated after insulin treatment^19^. Insulin, an essential hormone made by beta cells in the pancreas, lowers the level of glucose^77^. Insulin induces activation of mTORC1 and S6K1. The mTORC1-S6K1 axis is central to metabolic pathways, but the mechanisms downstream of this enzymatic axis are unclear. Arif et al. showed that the mTORC1-S6K1 axis in adipocytes stimulates EPRS1 phosphorylation at Ser999 and influences adiposity and aging (Fig. 2c)^19^. In this study, mice lacking S6K1 presented with reduced fat mass, delayed aging, and a longer healthy lifespan than WT control mice. The researchers found that EPRS1 was a key mediator of these physiological outcomes. To validate these effects, two mouse models were established by introducing a phosphorylation-resistant EPRS1 mutant harboring a serine-to-alanine (S999A) substitution or a phosphomimetic form harboring a serine-to-aspartic acid (S999D) substitution. The results were interesting: similar to S6K1-defective mice, mice harboring the S999A mutation exhibited lower body weight, reduced fat mass, and a longer lifespan (extended by 118 days) than WT mice. The authors found that insulin-stimulated lipid uptake was impaired in fat cells derived from S999A-mutant-harboring mice. When the S999D mutation in S6K1-deficient mice was complemented to restore EPRS1 phosphorylation, some of the fat mass was regained.

Notably, IFN-γ did not induce the phosphorylation of EPRS1 in mouse adipocytes or differentiated 3T3-L1 cells, meaning that GAIT was not assembled. Similarly, although insulin treatment of adipocytes induced EPRS1 phosphorylation at Ser999, it did not trigger assembly of the GAIT complex, possibly because L13a (a GAIT component) was not phosphorylated^19^. Further investigation into the underlying mechanism revealed that insulin-mediated free EPRS1 is bound to fatty acid transport protein 1 (FATP1). The Linker to EPRS1 was critical for this interaction, contributing in a phosphorylation-dependent manner^78^. Moreover, the phosphorylation-resistant S999A-mutant-containing Linker prevented FATP1 from binding EPRS1, but the EPRS1 S999D mutant interacted with FATP1 in the absence of insulin signaling. After binding FATP1, EPRS1 in adipocytes trafficked FATP1 to the plasma membrane. By translocating FATP1 to the membrane, EPRS1 facilitated long-chain fatty acid (LCFA) uptake, which increased the triglyceride synthesis rate (Fig. 3c). Knockdown of EPRS1 or FATP1 reduced the LCFA uptake rate, and knockdown of both proteins decreased LCFA absorption without impairing protein synthesis. Thus, EPRS1 phosphorylation contributes to lipid accumulation by inhibiting lipolysis and fatty acid oxidation^19^.

Another study suggested the possibility of targeting the S6K1-EPRS1 signaling axis to treat metabolic disease and possibly the pathology of aging^79^. Moreover, phosphorylation-defective EPRS1 model mice are useful for investigating many metabolic phenotypes, as well as the inflammatory activity and other noncanonical functions of EPRS1. However, the use of these model mice may confound the interpretation of diverse disease-related parameters. In addition, many questions must be answered to determine the mechanism through which the metabolic disease of S6K1-deficient mice was attenuated by the introduction of a single EPRS1 S999D protein, as S6K1 likely regulates multiple downstream targets. Whether an EPRS1 phosphorylation mutant can affect tissues in addition to adipocytes is another concern. In later studies, the same group showed that insulin-stimulated phosphorylation of EPRS1 at Ser999 by S6K1 required not only mTORC1 but also CDK5^51^. In addition to classical S6K1 phosphorylation at Thr389 by mTORC1, CDK5 was necessary for the phosphorylation of the S6K1 C-terminus at Ser424 and Ser429 (Fig. 2c). Multisite-phosphorylated S6K1 activated EPRS1 to regulate lipid metabolism. Moreover, multisite-phosphorylated S6K1 interacted with three additional lipid metabolism-related proteins (coenzyme A synthase, cortactin, and lipocalin 2) to control a posttranslational metabolon that affects adipocyte lipid metabolism. It is tempting to ask whether similar to EPRS1, these candidate proteins can confer metabolic and lifespan benefits to S6K1-deficient mice.

## Antiviral immune fuctions

Even after the noncanonical roles of ARSs had been discovered, their infection-related functions received little attention until the antiviral immune functions of EPRS1 were identified^18^. Because an MSCs functions as a stimulus-dependent depot system that responds rapidly to aberrant conditions, a research group hypothesized that MSCs regulate immune responses to viral infection. Large-scale RNA-Seq offers the opportunity to perform comprehensive and simultaneous transcriptional profiling during a host response to invading pathogens^80^. Taking advantage of this technique, researchers generated transcriptome profiles of MSCs in influenza virus-infected primary human bronchial epithelial cells. Both heterogeneous expression and temporal fluctuation in the expression of genes encoding MSC components were observed in response to the viral infection. A luciferase-based assay showed that among all the MSC components, EPRS1 induced significant antiviral activity via the IFN-β gene promoter.

EPRS1 knockdown in macrophages increased the replication of RNA viruses (influenza H1N1/PR8 and vesicular stomatitis virus (VSV)), but not that of a DNA virus (herpes simplex virus). Consistent with these findings, EPRS1-deficient cells showed considerable attenuation of the production of antiviral cytokines such as IFN-β and IL-6 following RNA virus infection or treatment with the synthetic double-stranded RNA poly(I:C); however, these outcomes were not observed following infection with herpes simplex virus, indicating that EPRS1 positively regulates antiviral innate immune responses, specifically those directed against RNA viruses.

Moreover, the survival rate of heterozygous *Eprs1*^+/−^ mice intravenously challenged with VSV-Indiana virus was lower than that of WT mice. Viral titers in the brain and spleen of *Eprs1*^+/−^ mice were higher than those in WT mice, which accounted for the increased susceptibility of EPRS1-deficient mice to viral infection. A histological analysis also supported the finding that *Eprs1*^+/−^ mice carry higher viral loads and higher numbers of infiltrating inflammatory cells than their counterparts. Taken together, these data suggest that EPRS1 triggers intracellular innate immune responses against viral infection.

Through proteomic analysis, virus infection-specific phosphorylation of EPRS1 at Ser990 but not at Ser999 was identified (Fig. 2d). The virus infection-specific modification was verified in assays with an in-house polyclonal anti-pSer990 antibody. Virus (PR8) infection induced a low phosphorylation rate of EPRS1 Ser990 after 1 h, and this rate increased gradually up to 24 h. IFN-γ treatment did not induce the phosphorylation of EPRS1 at Ser990. Concomitantly, EPRS1 in virus-infected cells did not form the GAIT complex or suppress the expression of GAIT genes such as Cp^44^. These results strongly imply that Ser990 is a novel site of phosphorylation specifically after viral infection, enabling EPRS1 dissociated from MSCs to govern antiviral immune responses independent of the GAIT system.

Antiviral signaling is mediated mainly by retinoic acid-inducible gene 1 (RIG)-I-like receptor (RLR) pathways in which RIG-I and melanoma differentiation-association protein 5 (MDA5) sense RNA viruses, after which the two molecules are recruited to an adaptor protein called mitochondrial antiviral signaling protein (MAVS). MAVS then activates the type I IFN promoter and NF-kB^81^. Analysis of the RLR signaling cascade revealed that EPRS1 functions at the RIG-I/MAVS axis, which is central to antiviral signaling. An interactome analysis based on MAVS signaling revealed that EPRS1 interacted with poly(C)-binding protein 2 (PCBP2), a protein known to trigger the ubiquitination and degradation of MAVS^82^. EPRS1 prevented the interaction between PCBP2 and MAVS by competitively targeting the same PCBP2 domain as MAVS. Thus, EPRS1 blocked PCBP2-mediated MAVS ubiquitination and degradation, thereby maintaining strong antiviral immune responses (Fig. 3d).

Domain mapping of EPRS1 revealed that the region encompassing amino acids 169–196 (a flexible linking region between GST and EARS1) is critical for EPRS1 interaction with PCBP2 (Fig. 3d). Deletion of this linker region from EPRS1 abrogated its ability to activate the *IFNB* promoter. Combining the functional region (aa 169–196) with the cell-penetrating peptide Tat^83^, known as Tat-Epep, significantly increased the production of antiviral cytokines and reduced the viral replication rate in macrophages^18^. As this synthetic peptide was derived from a natural host protein, it may exhibit therapeutic benefits and a high safety profile. The RIG-I/MAVS axis functions as a central hub for immune signaling in response to most RNA viruses. Thus, RIG-I/MAVS signaling activation is a potential therapeutic strategy against viral infection and replication. Improving the design of EPRS1-derived peptides may result in a pan-antiviral peptide against RNA viruses such as SARS-CoV-2 that would function by promoting RIG-I/MAVS signaling and antiviral cytokine production.

## Cancer-associated functions

Cancer arises from altered gene expression, which inevitably leads to dysregulated protein synthesis^84^. A gene expression signature can indicate the quantitative integration of multiple oncogenic events, as well as be a hallmark of cancer^85^. Since ARSs are essential for protein synthesis, their expression can be expected to be upregulated in cancer cells. However, each ARS gene exhibits dynamic and distinctive expression patterns depending on the type of cancer^86^. For example, EPRS1 is highly overexpressed in pancreatic adenocarcinoma (The Cancer Genome Atlas; TCGA dataset: https://www.cancer.gov/tcga)^86^. Furthermore, EPRS1 is upregulated in breast cancer, and its overexpression is linked to an unfavorable clinical outcome^87^. Similarly, data from TCGA and METABRIC cohorts showed that EPRS1 was expressed at high levels in estrogen receptor-positive (ER^+^) breast cancer tissues and was related to reduced overall survival^88^. In addition, a transcriptome analysis revealed that EPRS1 regulated the cell cycle and estrogen response gene expression^88^. Collectively, these results suggest that EPRS1 is a critical regulator of cell proliferation, estrogen signaling, and the development of specific cancers.

Alternatively, EPRS1 contributes to tumorigenesis by interacting with several target proteins^12^. One non-MSC EPRS1-targeted protein is the neural-specific transcription factor Engrailed 1^89^. Engrailed 1 is overexpressed exclusively in basal-like breast cancer, and interference with its expression triggers potent and selective cell death. For example, Beltran et al. developed a synthetic interference peptide comprising specific Engrailed 1 sequences and found that the peptide affected downstream EPRS1 effectors in breast cancer cells via its interaction with EPRS1^89^. This interaction reduced the survival of basal-like breast cancer cells. In addition, EPRS1 interacts with tRNA-dihydrouridine synthase (Dus2) to increase translation efficiency. Dus2 upregulation correlated positively with pulmonary carcinogenesis and indicated a poorer prognosis for lung cancer patients^90^. Hence, the EPRS1-Dus2 interaction might effectively drive the proliferation of cancer cells, possibly by promoting tRNA charging activity^90^. Finally, a recent study reported that EPRS1 was often overexpressed in gastric cancer tissues and that its expression correlated positively with cancer development both in vitro and in vivo^91^. Mechanistically, EPRS1 interacted directly with SYCL2 to activate the WNT/GSK-3β/β-catenin signaling pathway and promote cell proliferation and tumor growth^91^.

Because prolonged inflammatory gene expression contributes to malignant tumor progression, GAIT-mediated translational control of inflammatory gene transcription may protect cells from inflammation and injury in the presence of persistent inflammatory stimuli^92^. In particular, the GAIT complex silences a key angiogenic factor, VEGF. Angiogenesis driven by VEGF promotes the transition of small tumors into large rapidly growing and vascularized tumors^93^. Therefore, GAIT-mediated translational silencing of VEGF may play a crucial role in protecting cells from tumorigenesis^44^.

In conclusion, therapeutic and diagnostic uses of EPRS1 are feasible. First, the upregulated expression of EPRS1 may be a diagnostic and prognostic (poor) biomarker for multiple cancer types, especially breast cancer^87^. Modulation of the EPRS1 interaction via its binding proteins may be another effective therapeutic strategy for preventing cancer development. For example, Engrailed 1-derived interference peptides combined with other specific inhibitors of EPRS1 may be extremely effective at inhibiting basal-like breast cancer^89^. In addition to direct EPRS1 inhibitors, investigation into potential antagonists that block EPRS1 phosphorylation and dissociation from MSCs (e.g., kinase inhibitors) are interesting candidates for application to cancer therapy.

## Other disease-related functions

Multiple single-nucleotide polymorphisms or point mutations in EPRS1 have been reported in patients. One study reported that biallelic mutations in *EPRS1* cause hypomyelinating leukodystrophy^94^. EPRS1 pathogenic mutations have been found in the PARS1 core domain. The study evaluated whether variants caused decreased protein availability, impaired tRNA synthetase function, and/or led to abnormal MSC assembly to decrease translation and protein production at a crucial time during brain development, resulting in deficient myelination. Immunoblotting, enzymatic activity assays, and mass spectrometric analysis revealed that EPRS-related leukodystrophy was related to abnormal protein production with or without abnormal aminoacylation but was unlikely to be related to MSC assembly^94^. Another report identified heterozygous variants in *EPRS1* in a 4-year-old patient presenting with psychomotor retardation, seizures, and deafness^95^. The mutated residues were identified in the EARS1 domain, which is conserved among species^95^. These studies reflect potential connections between EPRS1 and neurological diseases that are caused mainly by abnormalities in canonical tRNA aminoacylation.

Disease-associated compound heterozygous mutations in EPRS1 were also reported in two patients with diabetes and bone diseases^96^. These mutations lead to the amino acid substitutions P14R and E205G. Neither mutation affected tRNA binding or EPRS association with MSCs. Paradoxically, P14R, which is located in the noncatalytic GST domain and plays a critical role in MSC assembly, induced a conformational change that altered tRNA charging kinetics. E205G, located in the EARS1 domain, showed defective tRNA aminoacylation. This study also revealed that patient-derived cells expressing compound heterozygous EPRS1 showed heightened integrated stress responses, leading to disruption of protein homeostasis and reduced cell viability^96^. Future studies should be conducted to identify the mechanisms underlying disease phenotypes that correlate with mutation-driven changes in catalytic activity.

EPRS1 is linked closely to fibrosis. EPRS1 is overexpressed in failing human and mouse hearts. In cardiac fibroblasts stimulated with transforming growth factor-beta (TGF-β), increased expression of EPRS1 increased the translation rate of genes encoding proline-rich profibrotic proteins (e.g., collagens) via enhanced translation elongation^97^. Cardiac tissue-specific deletion of *Eprs1* in mice profoundly reduced cardiac fibrosis under pathogenic stress conditions. Halofuginone functions as an antifibrotic agent that binds the PARS1 domain in EPRS1^23,24^ and reduces its enzymatic activity^98^. Halofuginone treatment significantly decreased the translation efficiency of proline-rich collagens in cardiac fibroblasts and in TGF-β-activated myofibroblasts^97^. Halofuginone also inhibited the production of extracellular matrix (ECM) proteins such as procollagen and fibronectin both in vivo and in cultured fibroblasts^98^. However, the exogenous addition of proline or EPRS1 reversed this suppressive effect. In particular, halofuginone competitively inhibited proline in enzymatic assays^98^. Similarly, EPRS1 plays a role in idiopathic pulmonary fibrosis, a chronic disease-associated with abnormal accumulation of the ECM in fibrotic foci in the lung^99^. Authors showed that EPRS1 triggers TGF-β-mediated upregulation of ECM protein and mesenchymal marker expression. Additionally, EPRS1-dependent signal transducer and activator of transcription 6 (STAT6) phosphorylation induced ECM production in the lungs of bleomycin-treated mice. These results suggest that EPRS1 (particularly the PARS1 domain) is a potential drug target for the treatment of fibrotic diseases.

Technically, fibrosis is not a disease; in contrast, it is a condition that results from tissue injury and is associated with chronic inflammation and cancer^100^. The relationship between fibrosis and EPRS1 points to the potential functions of EPRS1 in diseases with a fibrotic outcome. Therefore, halofuginone may be used to inhibit inflammatory responses and cancer progression because it suppresses EPRS1 activity. Although no direct link between halofuginone-mediated inhibition of EPRS1 and cancer cell growth has been established to date, the action of halofuginone in reducing breast and prostate cancer bone metastases in mice (by inhibiting TGF-β/bone morphogenetic protein signaling) suggests the possibility that EPRS1 contributes to the underlying process^101^. Similarly, halofuginone suppressed inflammatory responses by preventing Th17 cell differentiation, which was also mediated via EPRS1 inhibition^102^. These data suggest that combination therapy (e.g., with halofuginone and other potential inhibitors that target ERPS1) may be beneficial for the treatment of fibrosis and associated diseases.

## Concluding remarks

There is a clear, strong tendency for evolution to add new sequences and domains to ARSs. The additions of these sequences are consistent with the involvement of ARSs in a broad range of biological functions in addition to protein synthesis and correlate with the increased biological complexity of higher organisms. Among human ARSs, EPRS1 is the most evolutionarily derived protein, and it is the only fusion tRNA synthetase with two different aminoacylation functions and a noncanonical translation-regulatory function that is mediated by the three repeated WHEP domain-containing linker region; however, it remains unclear why the two enzymes EARS1 and PARS1 are fusion partners. The fusion of genes encoding EARS1 and PARS1 to form EPRS1 presents an intriguing phenomenon. It has been observed that an increased demand for proline contributes to the marked depletion of glutamic acid, which in turn affects the cellular levels of glutamine and arginine^15^. EPRS1 plays a crucial role in regulating the steady-state levels of amino acids. Therefore, the gene fusion event is believed to have emerged from an ancient organism to ensure the coexpression and coregulation of both enzymes, thereby preventing lethal dysregulation^15^. The substantial evolutionary advantage is evident from the widespread expression of EPRS1 in nearly all extant animals. Moreover, the fusion of these two enzymes likely enhances the efficiency and coordination of protein synthesis, thereby contributing to organism survival and adaptation^14^.

EPRS1 resides in the outer portion of an MSC^4,44^. The location of multiple and selective phosphorylation sites (i.e., Ser886, Ser990, and Ser999) within the highly accessible linker region correlates with the rapid switching between the translational function of EPRS1 to the multiple biological functions in response to diverse stimuli (Fig. 2). EPRS1 is the first example of an ARS shown to effect on intracellular inflammatory and antiviral signaling pathways. After its discovery, researchers noticed that EPRS1 expression and subcellular localization were important for its pathophysiological function. Moreover, EPRS1 is a stimulus-dependent molecular switch that drives specific cell signaling pathways. Activation of EPRS1 by two different types of organismal pressure, i.e., inflammatory and metabolic stimuli, suggests central pluripotent roles in stress responses^78^. Considering its location in the MSC, it is still unclear how all these functions are mechanistically regulated. Due to its flexible nature, the structure of EPRS1 has not been solved. Unanswered questions regarding the structure and functions of EPRS1 may be addressed by integrated studies using key research technologies such as cryo-transmission electron microscopy and cross-linking proteomic analysis in combination with cellular and immunological assays. In conclusion, based on its multiple noncanonical functions in a stress- and interactor-specific manner (Table 1), EPRS1 shows great therapeutic and diagnostic potential in the context of many disorders, including infection, inflammation, metabolism-related immune dysregulation, and cancer.

**Table 1:** Phosphocodes, interactors, and functions of EPRS1 under different stresses.

Stimulus	Functional domain	Phosphorylation site	MSC association under stress	Interactor	Subcellular localization during function	Function	Disease	Refs.
IFN-γ	Linker	Ser886, Ser999	Dissociated	NSAP1, GAPDH and L13a for GAIT complex formation	Cytoplasm	Translational silencing of GAIT-element-containing inflammatory genes	Inflammatory disease	^10,13,16,17^
LPS, bacteria	EARS1, Linker, PARS1	Ser999	Dissociated	PARS1: AKT Linker: PI(3)P EARS1: Rab5	Early endosomes	Inflammation resolution	Inflammatory disease	^20^
Insulin	Linker	Ser999	Dissociated	FATP1	Plasma membrane	Adipocyte lipid metabolism	Obesity and aging	^19,79^
RNA virus	Residues 168-196	Ser990	Dissociated	PCBP2	Cytoplasm	MAVS-mediated antiviral immune function	Viral disease	^18^
Not defined	(Possibly) PARS1	Unknown	Not defined	Engrailed 1-derived interference peptide	Cytoplasm	Inhibiting cancer cell survival	Breast cancer	^89^
Not defined	Linker	Unknown	Not defined	DUS2	Cytoplasm	Increased translation efficiency	Lung cancer	^90^
Not defined	Not defined	Unknown	Not defined	SYCL2	Cytoplasm	Promoted tumor growth	Gastric cancer	^91^
Cardiac stress, TGF-β	PARS1	Unknown	Not defined	Proline for canonical activity	Cytoplasm	Increased collagen protein levels	Fibrotic disease	^97^
